# Evaluation of the Effect of Honey-Containing Chitosan/Hyaluronic Acid Hydrogels on Wound Healing

**DOI:** 10.3390/gels9110856

**Published:** 2023-10-28

**Authors:** Emine Şalva, Ahmet Enes Akdağ, Saadet Alan, Sema Arısoy, Fatma Jülide Akbuğa

**Affiliations:** 1 Department of Pharmaceutical Biotechnology, Faculty of Pharmacy, Inonu University, Battalgazi, Malatya 44210, Türkiye; 2 Department of Pharmaceutical Biotechnology, Faculty of Pharmacy, Marmara University, Başıbüyük, İstanbul 34854, Türkiye; enes.akdag@gmail.com; 3 Department of Medical Pathology, Faculty of Medicine, Inonu University, Battalgazi, Malatya 44210, Türkiye; saadet.alan@inonu.edu.tr; 4 Department of Pharmaceutical Technology, Faculty of Pharmacy, Selçuk University, Selçuklu, Konya 42250, Türkiye; sema.arisoy@selcuk.edu.tr; 5 Department of Pharmaceutical Technology, Faculty of Pharmacy, Medipol University, Beykoz, İstanbul 34815, Türkiye; fjakbuga@medipol.edu.tr

**Keywords:** chitosan, honey, hyaluronic acid, wound healing, hydrogel, tissue engineering

## Abstract

The 3D polymeric network structure of hydrogels imitates the extracellular matrix, thereby facilitating cell growth and differentiation. In the current study, chitosan/hyaluronic acid/honey coacervate hydrogels were produced without any chemicals or crosslinking agents and investigated for their wound-healing abilities. Chitosan/hyaluronic acid/honey hydrogels were characterized by FTIR, SEM, and rheology analysis. Moreover, their water content, water uptake capacities, and porosity were investigated. In FT-IR spectra, it was discovered that the characteristic band placement of chitosan with hyaluronic acid changed upon interacting with honey. The porosity of the honey-containing hydrogels (12%) decreased compared to those without honey (17%). Additionally, the water-uptake capacity of honey-containing hydrogels slightly decreased. Also, it was observed that hydrogels’ viscosity increased with the increased hyaluronic acid amount and decreased with the amount of honey. The adhesion and proliferation of fibroblast cells on the surface of hydrogel formulations were highest in honey-containing hydrogels (144%). In in vivo studies, wound healing was accelerated by honey addition. It has been demonstrated for the first time that honey-loaded chitosan-hyaluronic acid hydrogels, prepared without the use of toxic covalent crosslinkers, have potential for use in wound healing applications.

## 1. Introduction

Biodegradable polymers attract attention in tissue regeneration applications. For instance, natural polymers, including chitosan and hyaluronic acid, are frequently used in regenerative medicine and tissue engineering owing to their ability to form biocompatible hydrogels [1]. Hydrogels have matchless properties, such as high water-uptake capacity mechanically. Hydrogels composed of substances derived from the extracellular matrix (ECM) are used to engineer damaged tissues [2]. Hyaluronic acid (HA) is a polyanionic polysaccharide composed of repeated units of β-1,4-glucoronic acid and β-1,3 N-acetyl-d-glucosamine. HA promotes cell proliferation, adhesion, and differentiation as an important basic component of the ECM. Due to hyaluronidase enzyme activity, its half-life in tissues varies from a few hours to a couple of days [3]. Especially high-molecular-weight HA has high mucoadhesion properties in an acidic environment [4]. 

As a result of its good gelling properties, superior biodegradability, and biocompatibility, HA is widely used in wound healing. However, HA hydrogels degrade rapidly and have poor physical and mechanical properties. Therefore, it can be combined with polycationic chitosan to prolong the residence time of poly-anionic hyaluronic acid in the tissue and improve its physical properties [5]. Chitosan is a natural cationic polymer. The properties of chitosan depend on the deacetylation degrees and consist of glucosamine and N-acetyl-glucosamine units [6]. In tissue engineering, physically cross-linked chitosan hydrogels are prepared by the neutralization of chitosan. Chitosan hydrogels prepared using NaOH were stable at room temperature, biocompatible, and non-cytotoxic [7,8,9]. Studies have been conducted on various cell types, including stem cells, human endothelial cells, osteoblasts, keratinocytes, fibroblasts, and neuronal cells, to assess the biological properties of crosslinked chitosan hydrogels by neutralization [10]. Chitosan/HA hydrogels have been prepared using a complex coacervation method that utilizes non-specific electrostatic interactions between oppositely charged chitosan and hyaluronic acid polymers [11,12]. Chitosan is known to be an effective wound dressing material in a wound treatment approach [13]. The incorporation of various polymers, such as alginate, gelatin, and hyaluronic acid, has been found to augment the wound-healing characteristics of chitosan [14,15,16]. Chitosan/HA hydrogels protect wounds by creating a moist healing environment. Chitosan/HA hydrogels have been found to be particularly useful in cartilage treatment [17]. When chitosan and HA were combined, the resulting formulation increased cell proliferation and extracellular matrix production in chondrocyte encapsulation [18]. The number of publications on the use of HA in excisional wounds is limited. It was reported that HA with a high molecular weight induces angiogenesis and increases the rate of wound healing by protecting tissue integrity [4,19]. 

Commercial wound dressing materials containing high amounts of antimicrobial agents may delay wound healing due to their cytotoxic effects, especially in long-term treatments. In addition, their adhesion to the wound surface reduces the moisture level and damages the newly formed epithelium [20]. These problems can be overcome by using natural products that are effective in wound healing. Honey has been used effectively in traditional medicine for thousands of years for its antibacterial, anti-parasitic, and pain-relieving effects. Honey contains high concentrations of glycine, methionine, arginine, and proline, which are indispensable amino acids that facilitate collagen synthesis and the deposition of fibroblasts, especially during the wound healing process. Additionally, honey accelerates wound healing by creating a moist environment [21]. Therefore, it has been shown that hydrogels containing honey accelerate the wound healing process by supporting the humidification of the environment and contributing directly to tissue regeneration [22]. The antibacterial effect of honey, an essential parameter in wound healing, is due to its high osmolarity and low pH. Lower pH creates an environment that prevents bacterial growth, and the resulting acidic environment maximizes the oxygen required for wound repair [23]. Effective wound treatment can be provided with hydrogels prepared close to or similar to the skin pH. 

Although chitosan and HA hydrogels in wound healing have been reported before, there are not any studies for the chitosan/HA hydrogels containing honey. Another important aspect of this work is preparing hydrogels without using chemical crosslinkers. Chemical crosslinkers are commonly used to prepare biopolymeric hydrogels; however, chemically crosslinked hydrogels’ toxicity is a significant problem, limiting the biological applications of hydrogels [8,10]. Therefore, in this study, CS/HA/Honey hydrogel formulations were developed, and in vitro characterization and cell-culture studies were performed to evaluate the effects of chitosan/HA/honey coacervate hydrogels produced without the use of any chemical agent. In addition, its effects on wound healing were examined in detail in a rat wound model.

## 2. Results and Discussion

### 2.1. In Vitro Characterization of Chitosan/HA/Honey Hydrogels 

#### 2.1.1. Viscosity

**Figure 1:**
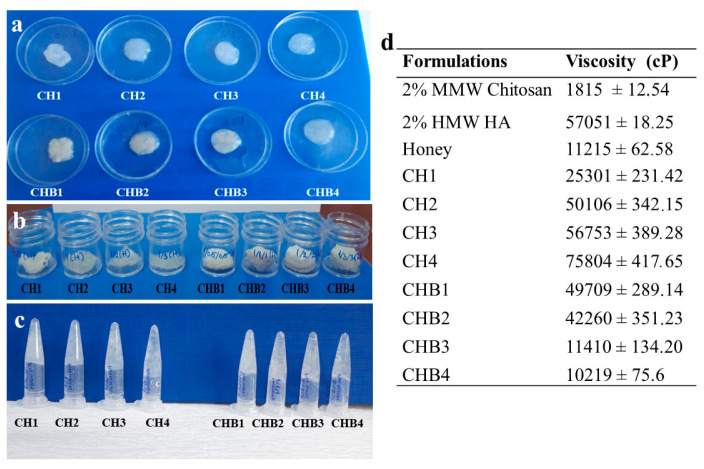
The viscosity and structure of hydrogel formulations. (**a**). Macroscopic images of different hydrogel formulations (**b**). Liyophilized hydrogels (**c**). Inversion test (**d**). Viscosity measurement values.

The chitosan/HA/honey formulations were visually examined for their color, integrity, and homogeneity (Figure 1a). It was observed that hydrogels have an opaque and homogeneous structure. Hydrogel-loaded eppendorf tubes were turned upside down to observe their flow against gravity. All formulations except CHB4, with the highest honey content, remained steady against gravity. CH3 and CHB3 formulations were ideal in terms of color, integrity, homogeneity, and spreadability (Figure 1c). 

The viscosity of the hydrogels affects the penetration of active ingredients through the skin. It was observed that viscosity increased with enhanced HA amount in the formulation composed of chitosan and HA (from 25,301 to 75,804 cP), while honey addition to the formulation decreased viscosity (from 49,709 to 10,219 cP) (Figure 1d). El Kased et al. [22] showed that the viscosity of hydrogels decreases with increasing concentrations of honey in the honey-chitosan or honey-carbopol 934 formulations. This shows that hydrogel formulations can flow easily from containers, and furthermore, the diffusion of honey within the hydrogel network would increase, resulting in a more effortless flow. Asfour et al. [24] reported that the positive correlation between viscosity and chitosan concentration led to an acceleration of the wound healing process due to the high viscosity of the formulation, resulting in sustained drug release with enhanced remains on the wound. 

#### 2.1.2. Morphology and Porosity

**Figure 2:**
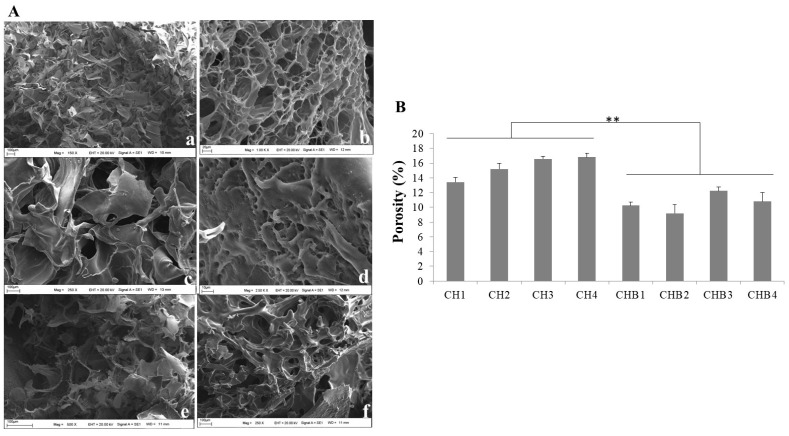
(**A**). SEM images of hydrogels. (**a**). Hyaluronic acid (**b**). Chitosan, (**c**). CH1, (**d**). CHB1, (**e**). CH3, (**f**). CHB3 hidrojelleri, (**B**). Porosity determination of CH and CHB formulations (** *p* = 0.002).

The scanning electron microscopy (SEM) technique was used to analyze the morphology of lyophilized hydrogels. The hydrogels that only consisted of chitosan exhibited a consistent pore structure, whereas the hydrogels that only consisted of HA displayed an irregular pore geometry, as depicted in Figure 2A(a,b). Chitosan/HA hydrogels at a 1/0.5 ratio (CH1) did not exhibit homogeneous pore structure due to the presence of HA. A flat surface morphology was observed in chitosan/HA/Honey (CHB1) hydrogels (Figure 2A(c,d)). In hydrogels with 1/2 (CH3) and 1/2/2 (CHB3) ratios, an increase in pore structure was observed with the increase in HA, as depicted in Figure 2A(e,f). Correia et al. [25] discovered chitosan scaffolds containing HA for cartilage tissue repair and showed that pore size increased with increasing HA concentrations. It has been observed that scaffolds consisting only of chitosan had a narrow pore configuration, and a broader pore structure was obtained with HA addition. Chitosan/HA scaffolds were reported to have high porosity and a regularly dispersed pore structure. Coimbra et al. [26] prepared HA/CS scaffolds by a polyelectrolyte complexation method, which showed a porous structure and interconnected pores. However, as HA increased, it was found that there was a closed and collapsed pore structure where the pores became irregular. As shown in Figure 2A, chitosan hydrogels were observed to form regular porous structures, while the addition of HA and Honey caused irregularities in the porous structure. However, for CHB3 formulations, it was observed that the porous structure was significantly preserved. 

Porous structures of hydrogels and scaffolds are essential for cell proliferation because they promote cell migration and the effective transport of nutrients and metabolic wastes [15]. In our study, the porosity value obtained by the solvent replacement method of the hydrogel formulations was found to be compatible with the SEM images (Figure 2). It was observed that the porosity in formulations containing honey was lower than in those without honey (Figure 2B). An increase in porosity was observed with the increase in HA concentration in the formulations (13–16%). It was determined that the porosity decreased with the addition of honey to the hydrogel formulation (1% 0). Dhasmana et al. [27] prepared a cell-free dermal matrix incorporating honey and showed that as the honey concentration increased, the pore size decreased in porosity measurements. 

#### 2.1.3. Swelling Ratio

**Figure 3:**
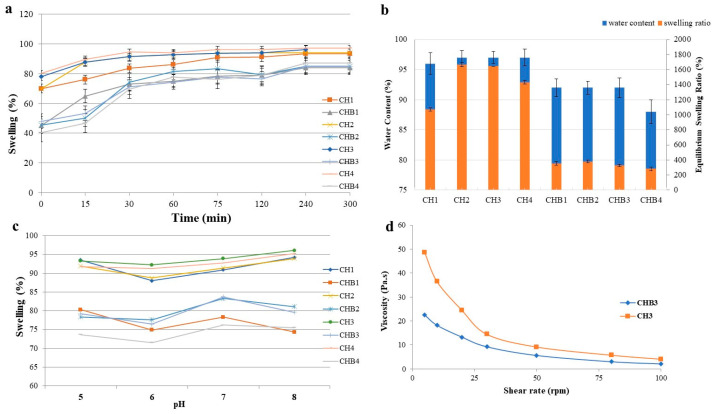
(**a**). % swelling ratios of hydrogels; (**b**). % water content and equilibrium swelling ratio; (**c**). % swelling ratios of hydrogels at different pHs; (**d**). Rheologic properties of CH3 and CHB3 formulations.

The porous structure of hydrogels and a proper swelling ratio allow oxygen diffusion through the hydrogel matrix, eliminate wound exudates, and maintain the moisture of the wounded area to contribute to wound healing [15]. An ideal wound dressing material should keep water loss under control and prevent factors such as leakage and dehydration as much as possible [28]. Chitosan and HA have numerous hydrophilic groups, such as hydroxyl, amino, and carboxyl, which contribute to hydrogel’s water uptake [25]. In our study, it was observed that formulations containing HA had enhanced water uptake capacity. The ratio of chitosan to HA significantly affected the swelling properties of hydrogels (Figure 3a). It was observed that the swelling ratio was decreased by honey addition to chitosan/HA formulations (Figure 3b). Zhu et al. [29] designed chitosan/HA hydrogels crosslinked with glycerol phosphate and observed that the swelling ratio increased in parallel with the amount of HA in the formulation. El-Kased et al. [22] reported that the swelling ratio of hydrogels varied inversely with the concentration of honey added to the formulation.

In formulations containing chitosan/HA and chitosan/HA/honey, it was observed that the swelling ratio of the hydrogels changed depending on the pH (Figure 3c). The highest swelling ratio in honey-containing formulations was observed at pH 7. In formulations containing chitosan/HA, the swelling ratio increased at pH 8. In hydrogels, the swelling rate increased as the ambient pH increased. The pH-sensitive swelling of hydrogels varies depending on the pH of the swelling medium, polymer concentration, hydrophilicity, ionizable groups, and the positive and negative charge-bearing groups of cationic and anionic polymers. The swelling of chitosan at an acidic pH is attributed to the protonation of its amino groups. Chitosan hydrogel swells as protonated, positively charged groups of the polymer chain cause repulsion. Anionic hydrogels based on hyaluronic acid swell at a higher pH because of the ionization of acidic groups. Ionized, negatively charged carboxyl groups in the polymer chain cause electrostatic repulsion and swelling [30].

The addition of HMW-HA (High Molecular Weight Hyaluronic Acid) to chitosan has increased viscosity, and the non-proportional nature of this increase indicates that HMW-HA exhibits non-Newtonian flow behavior. Furthermore, the addition of honey to the formulations has reduced viscosity in all groups. When examining the rheological properties of chitosan/HA and chitosan/HA/honey hydrogels prepared with high-molecular-weight hyaluronic acid, it is observed that the relationship between shear stress and shear rate conforms to a non-Newtonian flow model. As shown in Figure 3d, viscosity versus shear rate curves have been plotted for non-Newtonian flow-observed hydrogels. It is evident that as the shear rate increases, the viscosity of the hydrogels decreases, indicative of shear-thinning behavior. 

#### 2.1.4. FT-IR Analyses

**Figure 4:**
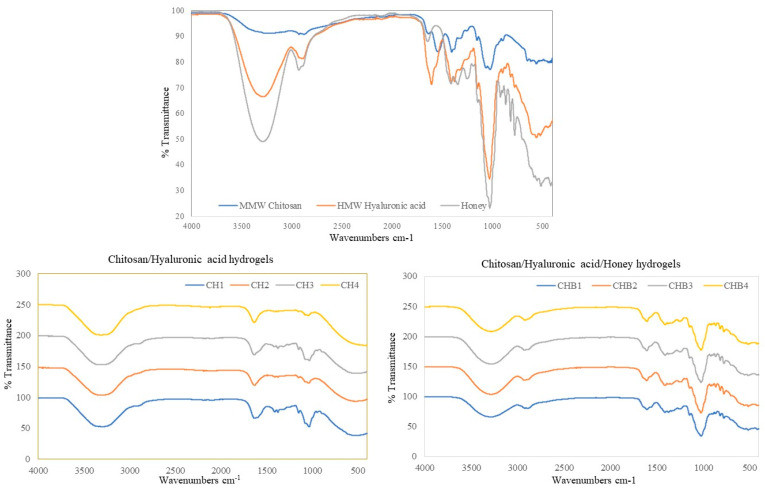
FT-IR measurements of hydrogels.

FTIR spectroscopy was performed for the identification of intermolecular interactions in chitosan/HA and chitosan/HA/honey hydrogels (Figure 4). Functional groups of MMW chitosan, HMW HA, and coacervate hydrogels are characterized. The characteristic peaks of chitosan were in the amide I band at 1647 cm^−1^, -NH_2_ bending vibrations at 1595 cm^−1^, and the amide III band at 1377 cm^−1^. The characteristic peak of hyaluronic acid is the C=O stretching vibration at 1640 cm^−1^ due to the carbonyl group. In chitosan/HA hydrogel formation, -NH_2_ bending vibrations at 1595 cm^−1^, an amide III band at 1377 cm^−1^ of chitosan, and C=O peaks at 1640 cm^−1^ of HA were observed. The characteristic peaks of honey (caused by glucose and fructose) are C-H and C=O, with C-O stretching bands at 2910 cm^−1^,1650 cm^−1^, and 1054 cm^−1^, respectively. The broad band at 3300 cm^−1^ comprises O-H stretching vibrations caused by sugars bonded with water. The FTIR spectrum of honey-containing formulations revealed band profiles similar to the spectrum of honey.

#### 2.1.5. Cell Attachment of Hydrogels, Cell Viability, and Proliferation

**Figure 5:**
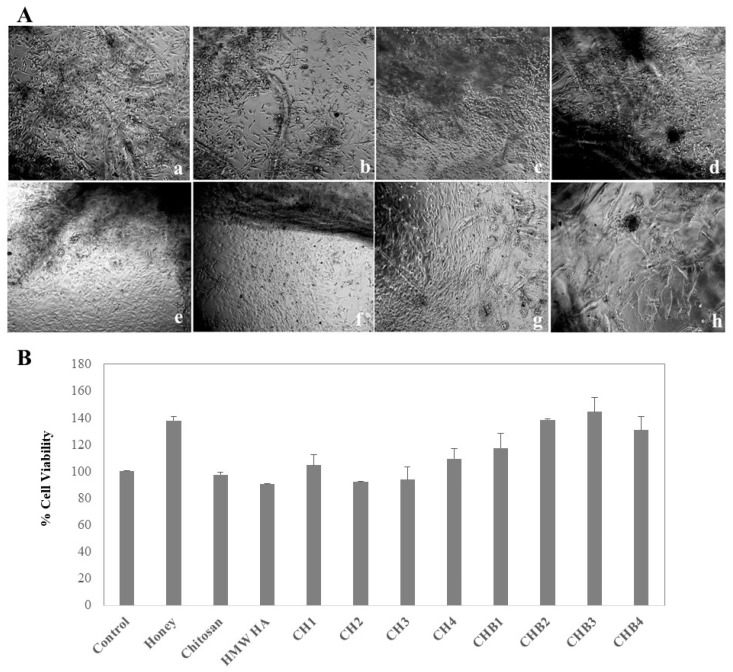
(**A**). Microscopic images of NIH-3T3 cells seeded on hydrogels (**a**–**d**: CH1, CH2, CH3, CH4; **e**–**h**: CHB1, CHB2, CHB3, CHB4 (20x magnification). (**B**). Cell viability by MTS test.

The adhesion and growth of fibroblast cells to the hydrogel surface were examined by reversed-phase microscopy. In all groups, it was determined that fibroblast cells adhered to the hydrogel surface and increased cell proliferation after 72 h (Figure 5). The cells continued to grow on and around the hydrogel, and no signs of cell damage, such as cell fragmentation, were observed. Thus, it was observed that hydrogels did not show any cytotoxic effect on the cells (Figure 5A,B). Although the cells grew toward direct contact with dense hydrogel sites, they did not migrate to these sites. Cells migrated to areas where the hydrogel was thin. It is difficult to identify proliferating cells in hydrogels because the cells do not have enough space to spread, so they grow into the hydrogel (Figure 5A). In a study, fibroblastic cells were implanted on hydrogels prepared with chitosan/β-GP/HA and similar results were observed. In that study, it was reported that, owing to the high density of hydrogels, the cells grew around the hydrogel and did not show any toxic effects [31].

The effects of hydrogels on cell viability were evaluated in all groups, and it was observed that hydrogels were non-toxic and increased cell proliferation (Figure 5B). Cell proliferation was higher in chitosan/HA/honey hydrogels applied well than in control ones. The proliferation of fibroblast cells on the surface of hydrogels was highest in honey-containing hydrogels (144%). In a study designed by Al-Jadi et al. [32], cell viability in groups containing honey showed a 35% increase, depending on the concentration, compared to the control group. 

#### 2.1.6. In Vivo Wound Healing

**Figure 6:**
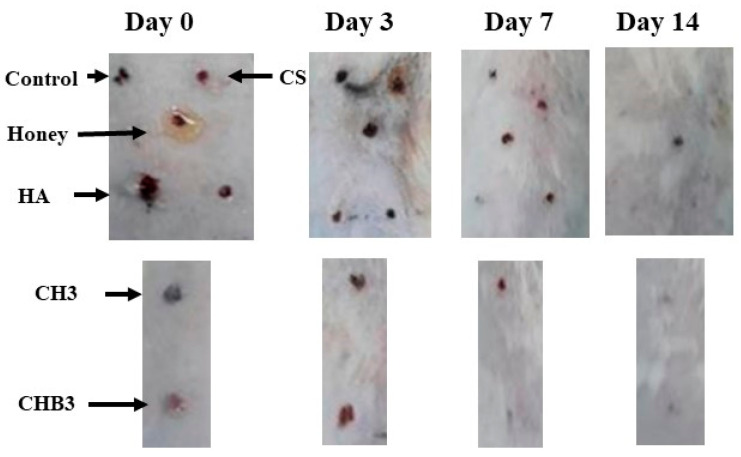
Macroscopic images of wound healing in all groups on days 0, 3, 7, and 14. Complete healing and wound closure occurred on the 21st day. Therefore, it is not included in the figure (six rats from each group were sacrificed on days 7, 14, and 21).

**Figure 7:**
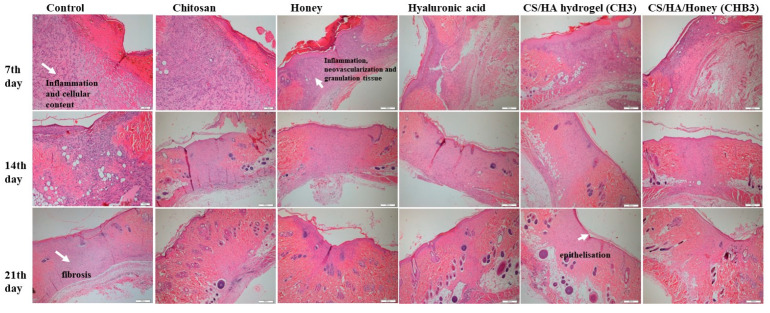
Histomorphological analysis of wound healing study. Microscopic examination of rat wound sites by H&E staining.

The process of wound healing is a multifaceted and systematically ordered phenomenon that involves several stages, including homeostasis, inflammation, cell migration and proliferation, and remodeling [33]. Macroscopic analysis of wound healing was shown in Figure 6. It was observed that CS/HA/Honey hydrogel application accelerated wound healing on the 7th day more than other groups. Complete closure of the wound occurred on the 21st day in all groups. Histopathological examinations were performed on days 7, 14, and 21 to evaluate the wound healing process (Figure 7). Interactions between keratinocytes and other cell types during wound healing are essential for complete wound closure. An imbalance in cellular or molecular mechanisms leads to fibrosis [34]. In our study, cellular content was found to be high in the groups where honey and honey-containing hydrogels were applied (Figure 7). Honey influences cellular responses such as cell migration and proliferation, collagen matrix production, and the chemotaxis of keratinocytes, fibroblasts, and endothelial cells. Therefore, honey may accelerate wound closure with re-epithelialization [35]. Chitosan has been shown to increase cell migration and cellular content in honey-containing wound care formulations [36]. 

In this study, wound samples were evaluated in terms of inflammation, and it was observed that the inflammation was relatively high in the group where only honey was applied on the 7th day. In the hydrogel-treated groups, inflammation was significantly higher on day 7 and decreased on day 14. Honey induces leukocytes for cytokine release, providing rapid autolytic debridement, and may increase the wound healing rate by suppressing inflammation [21,35].

The acidic composition of honey triggers the stimulation of fibroblasts and the expression of vascular endothelial growth factor (VEGF) [21]. In our study, we observed that hydrogel formulations containing honey significantly increased neovascularization on day 7 and decreased neovascularization on day 14 compared to other groups (Figure 7). Generally, endothelial cells participate in angiogenesis in tissue regeneration, and tissue repair facilitates the formation of new tissue and vessels. Deng et al. [34] reported that fibroblasts and endothelial cells increased in wounds treated with chitosan/HA hydrogels, resulting in faster tissue formation. 

Honey increases re-epithelialization, one of the most critical phases of wound healing [21]. In our study, epithelialization started on the 7th day, and it was observed that it was higher in the honey-containing hydrogel group compared to the other groups (Figure 7). Honey has been reported to accelerate epithelialization in wounds treated with chitosan/honey hydrogels [22]. Hydrogels cross-linked with PVP/PEG/honey have been shown to significantly accelerate dermal repair and increase re-epithelialization when used as a burn wound dressing [23]. Pectin-honey hydrogels significantly increased angiogenesis, matrix formation, granulation tissue formation, and re-epithelialization [37]. It seems that previous research supports the findings of our study. 

Hydrogels mimic natural tissue, or ECM, and promote angiogenesis. Chitosan/HA hydrogels were reported to be beneficial in the distribution of vascularization [34]. In our study, vascularization increased in the hydrogel-treated groups on day 7, while it decreased on day 14. On the 21st day, vascularization continued in the control and other groups. While granulation tissue formation increased on the 7th and 14th days in the hydrogel-applied groups and decreased after the 14th day, it continued to increase on the 14th day only in the chitosan and HA-applied groups and the control group (Figure 7). 

In the final phase of the repair process, fibroblasts continue to produce collagen and other ECM components, enabling the immature collagen matrix to be remodeled into mature scar tissue [34]. Insufficient accumulation of collagen leads to a weakening of the tissue structure. In our study, collagen synthesis in hydrogel groups continued until day 21. 

## 3. Conclusions

When honey is applied topically to the wound site, its rapid clearance is observed as a result of flowing from the wound site, which prevents it from reaching the therapeutic concentration by staying in the wound area for a long time. The chitosan/hyaluronic acid/honey hydrogels ensured that honey stayed longer in the wound area and showed its effect for a longer time. Effective wound healing has been achieved with the synergistic effects of chitosan, hyaluronic acid, and honey. We suggest that chitosan/hyaluronic acid/honey coacervate hydrogel formulations could be used as a potential therapeutic strategy for tissue regeneration. 

## 4. Materials and Methods

### 4.1. Materials

Chitosan M (Medium molecular weight with 190–310 kDa, 200–800 cP viscosity values with 75–85% deacetylated, Sigma, Tokyo, Japan), HA (high molecular weight with 1800 kDa, cosmetic grade, China), and honey (Balparmak, Türkiye) were used. Dulbecco’s modified eagle’s medium (DMEM), Fetal bovine serum (FBS), Trypsin-EDTA solution, and Dulbecco’s phosphate buffered saline (DPBS) were purchased from Gibco. MTS was purchased from Promega.

### 4.2. Methods

#### 4.2.1. Preparation of Chitosan-Hyaluronic Acid-Honey Hydrogels

**Figure 8:**
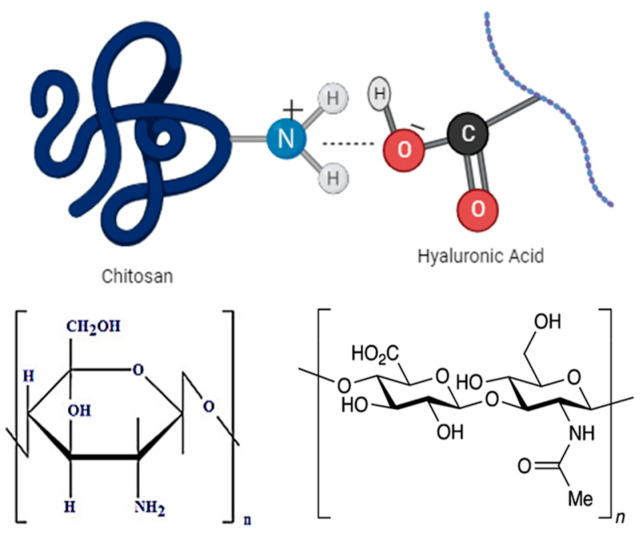
Chemical structures and interactions of chitosan and hyaluronic acid.

Chitosan/Hyaluronic acid/Honey coacervate hydrogels were prepared with high molecular weight hyaluronic acid and honey in different ratios (Table 1, Figure 8). Two percent medium molecular weight chitosan (MMW) was dissolved in 1% acetic acid (pH 3.5–4). NaOH was added to the chitosan solution drop by drop using a magnetic stirrer to raise the pH of the solution to 8–9 in order to precipitate chitosan. The resultant hydrogel was then centrifuged to remove excess NaOH and washed three times with distilled water. Aqueous solutions of high molecular weight hyaluronic acid were added to the precipitate at different concentrations (1%, 2%, 4%, and 6%) and mixed for one hour in a magnetic stirrer to form chitosan-hyaluronic acid hydrogel (pH 7.5–8.0) [2,38]. Honey (1%, 2%, 4%, and 6%) was added to this hydrogel in the same weight ratio as hyaluronic acid, and stirring the hydrogel continued (pH 6.5–7.0). 

#### 4.2.2. Viscosity Study

Viscosity determination of Chitosan/HA and Chitosan/HA/Honey hydrogels was performed at 25 °C at a shear rate from 1 to 100 rpm (Rheocalc TV1.0.1, Brookfield). Viscosity values were read after 2 min. Viscosity measurements (*n* = 6) were averaged and recorded.

#### 4.2.3. Morphology Study

Lyophilized hydrogel samples were coated onto aluminum grids coated with carbon film for scanning electron microscopy (SEM, Jeol, Tokyo, Japan), and the SEM samples prepared in this way were magnified at ×200–500 ratios with an accelerating voltage of 20 kV. 

#### 4.2.4. Porosity Measurements of Hydrogels

A solvent replacement method was used to define the porosity of hydrogels [39]. One gram of the hydrogel was weighed in small plastic petri dishes. The weighed hydrogels were dried until they reached a constant weight in an oven at 37 °C, and porosity tests were performed. The dry formulas were soaked in absolute ethanol overnight, and the next day, excess ethanol was removed from the environment by absorbent paper and weighed. Porosity was calculated according to the following formula. 

Porosity = [(A2 − A1)/(p.V)] × 100 A1 = Weight of hydrogel after immersion in absolute ethanol; A2 = Weight of hydrogel before immersion in absolute ethanol; *p* = Density of absolute ethanol; V = Volume of hydrogel.

#### 4.2.5. Determination of Water Content

To determine the water content of the prepared hydrogels, 1 g of each hydrogel was carefully weighed in small plastic petri dishes. The weighed samples were dried until they reached a constant weight in the oven at 37 °C. The dried hydrogels were weighed using a precision balance. They were then submerged in PBS until they reached equilibrium, and the swollen hydrogels were weighed after removing the excess with a filter paper. The swelling ratio and equilibrium water content were calculated using the following equations [40]:

Water content (%) = (Wwet – Wdry)/Wwet × 100

Swelling ratio (%) = (Wwet – Wdry)/Wdry × 100

Wdry is the weight of dried hydrogel, and Wwet is the weight of fully swollen hydrogel after reaching equilibrium. The degree of swelling and water content were calculated by taking an average of three measurements. 

#### 4.2.6. Swelling Control

One gram of each hydrogel formulation was weighed in a small plastic petri dish. The weighed hydrogels were dried in the oven at 37 °C until they reached a constant weight, and then swelling experiments were performed. Approximately 0.5 mL of PBS buffer was added to the hydrogels at 15 min intervals. At the end, excess buffer was removed from the environment with absorbent paper, and the hydrogels were weighed. The water holding capacity (percentage of swelling) of the formulations was calculated according to the formula below [41]:

% Swelling Ratio = (Wn – W0)/Wn × 100

Wn = *n*. weight of hydrogel sample per minute

W0 = Weight of the hydrogel sample at 0 min (start point).

#### 4.2.7. Ph Sensitivity Study

The Ph sensitivity of hydrogels was studied using PBS at different Ph levels (Ph 5–8) [42]. A total of 1 g of each hydrogel formulation was weighed in a small plastic petri dish. The weighed hydrogels were dried in the oven at 37 °C until they reached a constant weight, and then pH sensitivity tests were performed. In this study, 0.5 mL of PBS buffer was added to the hydrogels at 15 min intervals. At the end of the period, excess PBS was removed with absorbent paper, and the hydrogels were weighed. Dried hydrogels continued to be weighed at certain time intervals until swelling reached an equilibrium state. Swelling rates were calculated according to the formula shown below:

Swelling ratio = (Wn − W0)/W0

Wn = *n*. weight of hydrogel sample per minute, 

W0 = Weight of the hydrogel sample at 0 min (start point).

#### 4.2.8. Characterization of Hydrogels by Fourier Transform Infrared Spectroscopy

In order to investigate the bonding between polymer hydrogel formulations, hydrogel samples were lyophilized by freezing at −80 °C and analyzed using a Fourier Transform Infrared Spectroscopy method. Lyophilized hydrogel sample spectra were taken using a Perkin Elmer 1600 FT-IR device. 

#### 4.2.9. Adhesion of Cells to Hydrogels and the Effect on Proliferation

The NIH-3T3 cell line, which is a mouse fibroblast cell, was utilized in the cell-culture investigations of hydrogel formulations. The cells were cultured in a DMEM medium that was supplemented with 10% fetal bovine serum, 100 mM L-glutamine, and 100 mM antibiotic solution. Hydrogels were produced using aseptic techniques for the purpose of adhesion analysis. These hydrogels were subsequently placed in 12-well culture plates within laminar flow cabinets, sterilized using UV light, and left to dry overnight. On the subsequent day, NIH-3T3 fibroblast cells were seeded onto hydrogels at a density of 1 × 105 cells per well, and the adhesion of the cells to the surface was evaluated. 

The MTS assay was conducted to investigate the impact of hydrogels on cellular proliferation and cytotoxicity. DMEM with 10% FBS was added to the hydrogels that were sterilized and dried in 96 well-plates. To determine cell viability, 1 × 104 NIH-3T3 cells per well were added to hydrogels. After 72 h, cell viability was examined by the MTS method. The MTS method was carried out according to the manufacturer‘s protocol. At the end of the incubation period, 20 μL of MTS agent was added to the wells and incubated in the cell culture incubator for three hours. Cell viability was quantified by measuring absorbance at 490 nm using a Synergy plate reader (BioTek, Charlotte, VT, USA). 

#### 4.2.10. In vivo studies

Twelve-week-old Sprague Dawley rats (280–300 g) were supplied from the Inonu University Experimental Animals Laboratory. In vivo studies were performed in accordance with institutional regulations and the ethics committee for the use of laboratory animals (Inonu University, Malatya, Türkiye, 2019/A-28). 

After general anesthesia, the back hair of the rats was shaved and wiped with povidone. A full-thickness wound (containing the epidermis and dermis) using a biopsy instrument with a 6 mm diameter was inflicted on each side of the animals’ dorsal region. The rats were divided into five groups, with six rats in each group: control (saline), chitosan, honey, HA, chitosan/HA (1/2, CH3), and chitosan/HA/honey (1/2/2, CHB3) hydrogel groups. After wound formation, hydrogels and honey were applied to the wounds every three days. The wound healing effect of chitosan/HA/honey hydrogels was measured histopathologically on days 7, 14, and 21. A piece of skin up to the muscle layer covering the wound and an area of 5–10 mm^2^ from the uninjured part around the wound were removed. Afterwards, a histopathological examination of skin tissues was performed. The effects of the formulations on wound healing were evaluated by examining parameters such as inflammatory cells, fibroblasts, epithelial migration, re-epithelialization, new capillary formation, and collagen fiber deposition [43]. 

#### 4.2.11. Statistical Analysis

All data are expressed as mean ± standard deviation. A student’s t test and one-way ANOVA were applied to calculate *p*-values and examine significant differences between experimental data, and *p* < 0.05 was considered significant. Statistical analysis was performed with SPSS version 12.0. 

## Figures and Tables

**Table 1:** The composition of hydrogel formulations.

Formulations	HMW Hyaluronic Acid	MMW Chitosan	Honey	CS/HA CS/HA/Honey (*w*/*w*)
CH1	1%	2%		1/0.5
CH2	2%			1/1
CH3	4%			1/2
CH4	6%			1/3
CHB1	1%		1%	1/0.5/0.5
CHB2	2%		2%	1/1/1
CHB3	4%		4%	1/2/2
CHB4	6%		6%	1/3/3

## Data Availability

Data will be made available on request.

## References

[1] Sun J.C., Xiao C., Tan H.P., Hu X.H. (2013). Covalently crosslinked hyaluronic acid-chitosan hydrogel containing dexamethasone as an injectable scaffold for soft tissue engineering. J. Appl. Polym. Sci..

[2] Vignesh S., Sivashanmugam A., Mohandas A., Janarthanan R., Iyer S., Nair S.V., Jayakumar R. (2018). Injectable deferoxamine nanoparticles loaded chitosan-hyaluronic acid coacervate hydrogel for therapeutic angiogenesis. Colloid. Surf. B.

[3] Kawano Y., Patrulea V., Sublet E., Borchard G., Iyoda T., Kageyama R., Morita A., Seino S., Yoshida H., Jordan O. (2021). Wound Healing Promotion by Hyaluronic Acid: Effect of Molecular Weight on Gene Expression and In Vivo Wound Closure. Pharmaceuticals.

[4] Snetkov P., Zakharova K., Morozkina S., Olekhnovich R., Uspenskaya M. (2020). Hyaluronic Acid: The Influence of Molecular Weight on Structural, Physical, Physico-Chemical, and Degradable Properties of Biopolymer. Polymers.

[5] Croisier F., Jérôme C. (2013). Chitosan-based biomaterials for tissue engineering. Eur. Polym. J..

[6] Aswathy S.H., Narendrakumar U., Manjubala I. (2020). Commercial hydrogels for biomedical applications. Heliyon.

[7] Fiamingo A., Montembault A., Boitard S.E., Naemetalla H., Agbulut O., Delair T., Campana-Filho S.P., Menasché P., David L. (2016). Chitosan Hydrogels for the Regeneration of Infarcted Myocardium: Preparation, Physicochemical Characterization, and Biological Evaluation. Biomacromolecules.

[8] Xu Y., Xia D., Han J., Yuan S., Lin H., Zhao C. (2017). Design and fabrication of porous chitosan scaffolds with tunable structures and mechanical properties. Carbohydr. Polym..

[9] Furuike T., Komoto D., Hashimoto H., Tamura H. (2017). Preparation of chitosan hydrogel and its solubility in organic acids. Int. J. Biol. Macromol..

[10] Pita-López M.L., Fletes-Vargas G., Espinosa-Andrews H., Rodríguez-Rodríguez R. (2021). Physically cross-linked chitosan-based hydrogels for tissue engineering applications: A state-of-the-art review. Eur. Polym. J..

[11] Kayitmazer A.B., Koksal A.F., Kilic Iyilik E. (2015). Complex coacervation of hyaluronic acid and chitosan: Effects of pH, ionic strength, charge density, chain length and the charge ratio. Soft Matter.

[12] Lalevée G., David L., Montembault A., Blanchard K., Meadows J., Malaise S., Crépet A., Grillo I., Morfin I., Delair T. (2017). Highly stretchable hydrogels from complex coacervation of natural polyelectrolytes. Soft Matter.

[13] Liu H., Wang C., Li C., Qin Y., Wang Z., Yang F., Li Z., Wang J. (2018). A functional chitosan-based hydrogel as a wound dressing and drug delivery system in the treatment of wound healing. RSC Adv..

[14] Wang T., Zhu X.-K., Xue X.-T., Wu D.-Y. (2012). Hydrogel sheets of chitosan, honey and gelatin as burn wound dressings. Carbohydr. Polym..

[15] Wang X., Xu P., Yao Z., Fang Q., Feng L., Guo R., Cheng B. (2019). Preparation of Antimicrobial Hyaluronic Acid/Quaternized Chitosan Hydrogels for the Promotion of Seawater-Immersion Wound Healing. Front. Bioeng. Biotechnol..

[16] Kibungu C., Kondiah P.P.D., Kumar P., Choonara Y.E. (2021). This Review Recent Advances in Chitosan and Alginate-Based Hydrogels for Wound Healing Application. Front. Mater..

[17] Sultankulov B., Berillo D., Sultankulova K., Tokay T., Saparov A. (2019). Progress in the Development of Chitosan-Based Biomaterials for Tissue Engineering and Regenerative Medicine. Biomolecules.

[18] Park H., Choi B., Hu J., Lee M. (2013). Injectable chitosan hyaluronic acid hydrogels for cartilage tissue engineering. Acta Biomater..

[19] Gao F., Liu Y., He Y., Yang C., Wang Y., Shi X., Wei G. (2010). Hyaluronan oligosaccharides promote excisional wound healing through enhanced angiogenesis. Matrix Biol..

[20] Negut I., Grumezescu V., Grumezescu A.M. (2018). Treatment Strategies for Infected Wounds. Molecules.

[21] Nezhad-Mokhtari P., Javanbakht S., Asadi N., Ghorbani M., Milani M., Hanifehpour Y., Gholizadeh P., Akbarzadeh A. (2021). Recent advances in honey-based hydrogels for wound healing applications: Towards natural therapeutics. J. Drug Deliv. Sci. Technol..

[22] El-Kased R.F., Amer R.I., Attia D., Elmazar M.M. (2017). Honey-based hydrogel: In vitro and comparative In vivo evaluation for burn wound healing. Sci. Rep..

[23] Mohd Zohdi R., Abu Bakar Zakaria Z., Yusof N., Mohamed Mustapha N., Abdullah M.N.H. (2012). Gelam (*Melaleuca* spp.) Honey-Based Hydrogel as Burn Wound Dressing. Evid. -Based Complement. Altern. Med..

[24] Asfour M.H., Elmotasem H., Mostafa D.M., Salama A.A.A. (2017). Chitosan based Pickering emulsion as a promising approach for topical application of rutin in a solubilized form intended for wound healing: In vitro and in vivo study. Int. J. Pharm..

[25] Correia C.R., Moreira-Teixeira L.S., Moroni L., Reis R.L., van Blitterswijk C.A., Karperien M., Mano J.F. (2011). Chitosan scaffolds containing hyaluronic acid for cartilage tissue engineering. Tissue Eng. Part. C Methods.

[26] Coimbra P., Alves P., Valente T.A., Santos R., Correia I.J., Ferreira P. (2011). Sodium hyaluronate/chitosan polyelectrolyte complex scaffolds for dental pulp regeneration: Synthesis and characterization. Int. J. Biol. Macromol..

[27] Dhasmana A., Singh L., Roy P., Chandra Mishra N. (2018). Honey incorporated antibacterial acellular dermal matrix for full-thickness wound healing. Ann. Biotechnol..

[28] Lin Y.-J., Lee G.-H., Chou C.-W., Chen Y.-P., Wu T.-H., Lin H.-R. (2015). Stimulation of wound healing by PU/hydrogel composites containing fibroblast growth factor-2. J. Mater. Chem. B.

[29] Zhu Y., Tan J., Zhu H., Lin G., Yin F., Wang L., Song K., Wang Y., Zhou G., Yi W. (2017). Development of kartogenin-conjugated chitosan–hyaluronic acid hydrogel for nucleus pulposus regeneration. Biomater. Sci..

[30] Rizwan M., Yahya R., Hassan A., Yar M., Azzahari A.D., Selvanathan V., Sonsudin F., Abouloula C.N. (2017). pH Sensitive Hydrogels in Drug Delivery: Brief History, Properties, Swelling, and Release Mechanism, Material Selection and Applications. Polymers.

[31] Vieira de Souza T., Malmonge S.M., Santos A.R. (2021). Development of a chitosan and hyaluronic acid hydrogel with potential for bioprinting utilization: A preliminary study. J. Biomater. Appl..

[32] Al-Jadi A.M., Kanyan Enchang F., Mohd Yusoff K. (2014). The effect of Malaysian honey and its major components on the proliferation of cultured fibroblasts. Turk. J. Med. Sci..

[33] van de Vyver M., Boodhoo K., Frazier T., Hamel K., Kopcewicz M., Levi B., Maartens M., Machcinska S., Nunez J., Pagani C. (2021). Histology Scoring System for Murine Cutaneous Wounds. Stem Cells Dev..

[34] Deng Y., Ren J., Chen G., Li G., Wu X., Wang G., Gu G., Li J. (2017). Injectable in situ cross-linking chitosan-hyaluronic acid based hydrogels for abdominal tissue regeneration. Sci. Rep..

[35] Majtan J. (2014). Honey: An immunomodulator in wound healing. Wound Repair Regen..

[36] Sarhan W.A., Azzazy H.M., El-Sherbiny I.M. (2016). Honey/Chitosan Nanofiber Wound Dressing Enriched with Allium sativum and Cleome droserifolia: Enhanced Antimicrobial and Wound Healing Activity. ACS Appl. Mater. Interfaces.

[37] Giusto G., Vercelli C., Comino F., Caramello V., Tursi M., Gandini M. (2017). A new, easy-to-make pectin-honey hydrogel enhances wound healing in rats. BMC Complement. Altern. Med..

[38] Hu M., Yang J., Xu J. (2021). Structural and biological investigation of chitosan/hyaluronic acid with silanized-hydroxypropyl methylcellulose as an injectable reinforced interpenetrating network hydrogel for cartilage tissue engineering. Drug Deliv..

[39] Shahid N., Erum A., Zaman M., Tulain U.R., Shoaib Q.U., Majeed A., Rasool M.F., Imran I., Alshehri S., Noorani B. (2021). pH-Responsive Nanocomposite Based Hydrogels for the Controlled Delivery of Ticagrelor; In Vitro and In Vivo Approaches. Int. J. Nanomed..

[40] Im O., Li J., Wang M., Zhang L.G., Keidar M. (2012). Biomimetic three-dimensional nanocrystalline hydroxyapatite and magnetically synthesized single-walled carbon nanotube chitosan nanocomposite for bone regeneration. Int. J. Nanomed..

[41] Şalva E., Akbuğa J. (2017). The effects to GM-CSF expression and fibroblast proliferation of pGM-CSF containing chitosan/PVP hydrogels. Marmara Pharm. J..

[42] Fan L., Yang H., Yang J., Peng M., Hu J. (2016). Preparation and characterization of chitosan/gelatin/PVA hydrogel for wound dressings. Carbohydr. Polym..

[43] Mukherjee D., Azamthulla M., Santhosh S., Dath G., Ghosh A., Natholia R., Anbu J., Teja B.V., Muzammil K.M. (2018). Development and characterization of chitosan-based hydrogels as wound dressing materials. J. Drug Deliv. Sci. Technol..

